# Management of common bile duct stones: a comprehensive review

**DOI:** 10.3389/fsurg.2025.1658784

**Published:** 2025-11-12

**Authors:** Xiaojun Wang, Zhifeng Li

**Affiliations:** Handan First Hospital, Handan, Hebei, China

**Keywords:** common bile duct stones, endoscopic sphincterotomy, laparoscopic common bile duct exploration, post-procedural pancreatitis, SpyGlass cholangioscopy, extracorporeal shock wave lithotripsy, artificial intelligence, personalized treatment

## Abstract

CBD stones impose significant morbidity and cost. This review compares the efficacy and safety of endoscopic sphincterotomy (EST) and laparoscopic common bile duct exploration (LCBDE) and summarizes emerging techniques and remaining controversies. While EST remains a cornerstone for rapid stone extraction, particularly in high-risk surgical candidates, its association with post-procedural pancreatitis and long-term sphincter dysfunction underscores the need for judicious patient selection. Conversely, LCBDE emerges as a compelling alternative, offering superior complete stone clearance rates for larger or complex stones, reduced post-procedural pancreatitis, and the unique advantage of direct biliary tree visualization. However, its technical demands and bile leak risk necessitate standardized training protocols and institutional expertise. The review further explores cutting-edge adjunctive therapies, including SpyGlass cholangioscopy for impacted stones, extracorporeal shock wave lithotripsy (ESWL) for large calculi, and artificial intelligence-driven procedural planning, which collectively herald a new era of precision medicine in biliary surgery. Notably, hybrid approaches such as intraoperative endoscopic retrograde cholangiopancreatography (ERCP) demonstrate promising outcomes in reducing hospitalization and postoperative complications, albeit requiring multidisciplinary team coordination. Despite these advances, critical knowledge gaps persist regarding long-term functional outcomes of EST, cost-effectiveness of emerging technologies, and optimal management of anatomically complex cases. The lack of robust randomized trials comparing EST and LCBDE in diverse patient cohorts limits contemporary guideline development, underscoring the urgent need for high-quality comparative effectiveness research. This review synthesizes current evidence to advocate for a personalized, algorithmic approach to CBD stones management, balancing procedural risks, stone characteristics, and institutional capabilities. By highlighting translational research opportunities and unmet clinical needs, it provides a roadmap for advancing minimally invasive biliary surgery while challenging the global hepatobiliary community to prioritize patient-centered innovation and rigorous outcomes research.

## Introduction

CBD stones are a prevalent biliary disorder with significant clinical implications. The global incidence varies, but it is estimated that CBD stones are present in 10% of patients with symptomatic gallstones (1). In a systematic review and meta-analysis, the pooled incidence of CBD stones in patients presenting with acute cholecystitis was 13.7% (95% confidence interval 11.8–15.9) (2). These stones can lead to a range of complications, such as cholangitis, pancreatitis, and obstructive jaundice, which can significantly impact a patient's quality of life and even be life-threatening. For example, acute cholangitis, often caused by CBD STONES, could progress to septic shock if not promptly treated. The Chinese REgistry Study on the Treatment of Cholecysto Choledocholithiasis (CREST Choles) aimed to address the lack of data on the clinical outcomes and economic burden of treating cholecysto-choledocholithiasis in China (3). Understanding epidemiology is crucial for developing appropriate screening, prevention, and treatment strategies.

The management of CBD stones is fraught with challenges. Recurrence of CBD stones remains a significant issue, with some studies reporting recurrence rates varying depending on the treatment method. For instance, in a study comparing different treatment approaches, the recurrence rate was found to be influenced by factors such as the type of stone and the treatment modality (4). Complications associated with CBD stones treatment can also be a major concern. Post-ERCP pancreatitis remains the most frequent adverse event; a 2023 Cochrane meta-analysis of 145 RCTs reported an overall incidence of 9.8%, rising to 14.7% in high-risk subsets (5). Anatomical variations further complicate the management of CBD stones. Variations in the biliary anatomy, such as the presence of a periampullary or juxta papillary duodenal diverticulum, can make endoscopic or surgical procedures more difficult (6). These variations may affect the success rate of procedures and increase the risk of complications, highlighting the need for careful pre-operative assessment and individualized treatment plans.

The management of CBD stones has witnessed a significant shift towards minimally invasive techniques, with EST and LCBDE being two prominent approaches. EST has evolved as a key endoscopic technique for CBD stones, allowing for the removal of stones by incision of the sphincter. LCBDE, on the other hand, offers a minimally invasive surgical option for treating CBD stones. A meta-analysis comparing the two techniques showed that LCBDE may have advantages in terms of certain outcomes. For example, in some studies, LCBDE was associated with a lower rate of post-procedure pancreatitis compared to EST (7). However, the choice between EST and LCBDE depends on various factors, including patient characteristics, stone size and location, and the expertise of the medical team. Understanding the comparative efficacy of these techniques is essential for optimizing patient care.

## Overview of common bile duct stones

### Etiology and classification

The etiology of CBD stones is multifactorial. Cholesterol stones are mainly formed due to supersaturated bile with cholesterol, often associated with factors such as obesity, high - fat diet, and certain genetic predispositions (8). Pigment stones, on the other hand, are more prevalent in patients with conditions like chronic hemolytic anemia or biliary tract infections. These stones can be further classified as primary or secondary. Secondary CBD stones originate from the migration of gallbladder stones into the common bile duct, while primary CBD stones form directly in the common bile duct. In a study comparing primary and secondary choledocholithiasis in cholecystectomy patients, those with primary choledocholithiasis were older, had a greater body mass index, and a larger extrahepatic bile duct diameter (9). Understanding etiology and classification is crucial as it can guide treatment decisions, such as the use of medical dissolution therapy for cholesterol stones.

### Clinical presentation

CBD stones can present in various ways, either asymptomatically or with a range of symptoms. Asymptomatic CBD stones are often incidentally detected during imaging studies for other conditions. However, when symptomatic, they can cause jaundice, cholangitis, and pancreatitis. Jaundice occurs due to the obstruction of bile flow, leading to elevated bilirubin levels in the blood. Cholangitis is characterized by inflammation of the bile ducts, often presenting with fever, abdominal pain, and jaundice. Obstructive jaundice occurs in 74.4%–82.1% symptomatic patients with CBD stones (10). Pancreatitis can result from the obstruction of the pancreatic duct by CBD stones. In a systematic review and meta-analysis, asymptomatic patients with choledocholithiasis were found to have a higher risk of post-ERCP pancreatitis compared to symptomatic patients (11). Recognizing the different clinical presentations is essential for timely diagnosis and appropriate treatment.

### Diagnostic modalities

Imaging plays a crucial role in the diagnosis of CBD stones. Ultrasound is often the first-line imaging modality due to its non - invasiveness and wide availability. However, its sensitivity for detecting CBD stones can be limited, especially for small stones or stones in the distal common bile duct. Magnetic resonance cholangiopancreatography (MRCP) provides detailed images of the biliary and pancreatic ducts, with a sensitivity of around 76.8% for detecting any abnormality in a pediatric population when compared to ERCP (12). ERCP is not only a diagnostic tool but also a therapeutic option, allowing for the direct visualization and removal of CBD STONES. However, it is associated with a risk of complications, such as post-ERCP pancreatitis. Endoscopic ultrasound (EUS) has shown high diagnostic accuracy for CBD stones, with a pooled sensitivity of 0.97 and specificity of 0.90 in a meta-analysis (13). The choice of imaging modality depends on factors such as the patient's clinical presentation, the suspected size and location of the stones, and the availability of the technology.

### Biochemical markers

Biochemical markers can provide valuable information in the diagnosis and management of CBD stones. Elevated bilirubin levels, especially conjugated bilirubin, are often seen in patients with CBD stones due to biliary obstruction. Persistent elevation of serum total bilirubin (>2 × ULN) in patients with CBD stones may indicate severe biliary obstruction and warrants further evaluation for complications (14). Alkaline phosphatase is another commonly used marker, and its elevation can indicate biliary obstruction. Elevated alkaline phosphatase and bilirubin levels in patients with CBD stones reflect biliary obstruction and correlate with disease severity (15). However, these markers are not specific to CBD stones and can be elevated in other biliary and liver diseases, so they need to be interpreted in conjunction with imaging and clinical findings.

## Approaches to CBD STONES management

### Traditional approaches to common bile duct stones management

#### Open surgical exploration

Open surgical exploration for common bile duct stones has a long - standing history in the treatment of biliary diseases. In the past, it was one of the primary methods to address common bile duct stones when other less invasive techniques were not available (16). However, this approach is associated with several significant limitations.

The morbidity associated with open surgical exploration is relatively high. For instance, a study analyzing patients who underwent mid-line laparotomy found that male gender and acidosis were associated with open abdominal treatment, and the open abdomen group had a mortality rate of 27% (17). In the context of common bile duct stone surgery, the invasive nature of the procedure can lead to various complications such as wound infections, bleeding, and damage to surrounding organs. Additionally, in patients undergoing open procedures for common bile duct stones, the extensive tissue dissection and potential for postoperative adhesions contribute to a longer hospital stay and a slower return to normal activities. Prolonged recovery is also seen in other open surgeries; for example, in patients undergoing open surgery for lumbar ligamentum flavum cyst, traditional open surgery was associated with a prolonged recovery time compared to endoscopic treatment (18).

#### Endoscopic retrograde cholangiopancreatography (ERCP)

ERCP has significantly evolved over the years and become a cornerstone in the diagnosis and therapy of common bile duct stones. Initially, it was mainly used for diagnostic purposes, allowing visualization of the biliary and pancreatic ducts. However, with technological advancements, it has transformed into a highly effective therapeutic modality (19).

In terms of diagnosis, ERCP can provide detailed images of the bile ducts, enabling the detection of stones, strictures, and other abnormalities. For example, in a study comparing the diagnostic performance of magnetic resonance cholangiopancreatography (MRCP) and ERCP in the pediatric population, ERCP was used as the reference standard, highlighting its importance in accurately diagnosing biliary and pancreatic abnormalities (12). In terms of therapy, ERCP is now the mainstay for the treatment of choledocholithiasis. A meta - analysis of studies comparing balloon and basket catheters for the extraction of stones ≤ 10 mm in size during ERCP found that balloon catheters had higher complete stone clearance rates than basket catheters (relative risk 1.1, confidence interval 1.03, 1.18, *P* = 0.006) (19). ERCP can also be used for biliary stenting in cases where stones are not immediately removable, such as in elderly or high-risk patients, providing a palliative solution (20).

### Endoscopic sphincterotomy (EST) & laparoscopic common bile duct exploration (LCBDE)

#### Endoscopic sphincterotomy (EST)

EST involves several procedural steps. First, the endoscope is inserted into the duodenum to locate the ampulla of Vater. Then, a sphincterotome is used to incise the sphincter of Oddi, which allows for the extraction of common bile duct stones. Over time, there have been significant advancements in the devices used for EST (21). One of the notable advancements is the development of different types of sphincterotomes, such as the needle-knife and pull-type sphincterotomy devices. A study comparing the needle-knife pancreatic sphincterotomy technique (NKS) and the standard pull-type sphincterotomy (PTS) found that the incidence of post-ERCP pancreatitis was not significantly different between the two techniques (6.4% for NKS and 7.8% for PTS) when routine prophylactic pancreatic duct stent placement was used (22). Another study comparing transpancreatic sphincterotomy and needle - knife sphincterotomy for difficult biliary cannulation showed that there was no significant difference in the initial and eventual success rates between the two methods (82.9% vs. 90.8% and 90.0% vs. 90.8%, respectively), and the overall incidences of complications and acute pancreatitis were also not significantly different (14.3% vs. 18.4% and 11.4% vs. 11.8%, respectively) (23). These findings suggest that both techniques can be effective, but the choice may depend on the specific situation and the endoscopist's experience. Surprisingly, EST achieves high initial stone-clearance rates (> 90%) for stones ≤ 10 mm (19), and the rate can be further improved to ≈ 97% when combined with balloon dilation for 10–20 mm stones (24).

Adjunctive techniques are often used in conjunction with EST to improve the treatment of common bile duct stones. Balloon dilation, such as endoscopic papillary balloon dilation (EPBD), can be used to enlarge the opening of the sphincter of Oddi, facilitating the extraction of larger stones. A study comparing EST followed by large balloon dilation (LBD) with EST followed by mechanical lithotripsy (ML) for the management of large bile duct stones (12–20 mm) found that the complete bile duct stone removal rate was 97.7% in the EST - LBD group and 91.1% in the EST - ML group (*P* = 0.36), and the post - procedure complication rate was lower in the EST - LBD group (4.4% vs. 20%, *P* = 0.049) (25). Mechanical lithotripsy is another important adjunctive technique. It is used to break large stones into smaller fragments that can be more easily removed. However, it has its own risks, such as basket and stone impaction, which can lead to complications like pancreatitis and cholangitis (26). Cholangioscopy-guided therapy, including cholangioscopy-directed lithotripsy, has emerged as a valuable technique for difficult-to-treat stones. A prospective single-center series evaluating the efficacy and safety of a new digital single-operator peroral cholangioscope to guide laser lithotripsy for complicated biliary stones achieved a 94% stone clearance rate over 1 median procedure (27).

EST short-term complications-pancreatitis (9.7%) (5), bleeding (26), and rare perforation-are well documented (26); pancreatitis risk increases with biliary stent placement (OR 4.2) (28). In the long-term, papillary stenosis can develop, which may lead to recurrent biliary problems. A long-term follow - up study of patients who underwent EST for choledocholithiasis found that patients had an increased risk of acute pancreatitis and cholangitis in the long - term compared to those not treated with EST. The hazard ratio for endoscopic sphincterotomy vs. cholecystectomy was 5.5 (95% CI 3.5–8.4) for cholangitis and 4.9 (95% CI 2.8–8.6) for pancreatitis (29). Stone recurrence is also a concern. A study analyzing factors associated with recurrent bile duct stones after EST found that factors such as the size (diameter) of the largest CBD stone found at first presentation, diameter of the CBD at the first examination, use of mechanical lithotripsy, and presence of difficult lithiasis were associated with recurrence (30).

#### Laparoscopic common bile duct exploration (LCBDE)

LCBDE can be performed using either the transcystic or transductal approach. The transcystic approach refers to entering the CBD through the cystic duct, whereas the transductal approach (also termed choledochotomy) involves a direct anterior incision in the CBD wall. The transcystic approach involves accessing the common bile duct through the cystic duct, which is less invasive as it avoids direct manipulation of the common bile duct. However, it is limited by the size and anatomy of the cystic duct. If the cystic duct is too small or tortuous, it may be difficult to pass instruments through it for stone extraction (31). The transductal approach, on the other hand, involves making an incision directly in the common bile duct. This allows for better access to the stones, especially in cases where the stones are large or there are multiple stones. Intraoperative cholangiography is an important part of LCBDE. It helps in visualizing the biliary tree, identifying the location of stones, and ensuring complete stone removal. A study analyzing the outcomes of LCBDE with intraoperative cholangiography found that it can help in detecting and treating common bile duct stones effectively. For example, in a study of patients with common bile duct stones detected during intraoperative cholangiography, efforts to clear the ducts were associated with a lower risk of unfavorable outcomes compared to taking no measures (32).

Choledochoscopy plays a crucial role in LCBDE. It allows for direct visualization of the common bile duct, enabling the identification of stones, assessment of the biliary anatomy, and confirmation of complete stone removal. For instance, in the case of a double gallbladder with a common bile duct stone, laparoscopic choledochoscopy was used to explore and remove the stone through the cystic duct, followed by a primary suture of the cystic duct without using a T-tube (33). Stone extraction tools such as baskets and balloons are essential for removing stones during LCBDE. Baskets are often used to grasp and retrieve stones, while balloons can be used to push stones into a more favorable position for extraction or to dilate the ampulla of Vater to facilitate stone passage. In CBD stones < 1.5 cm, basket and balloon catheters achieve similar stone-clearance success, but balloons are associated with a lower risk of bleeding or perforation (34). In CBD stones < 2 cm, the complete clearance rate was slightly higher with balloon catheters (96.3% vs. 91.7%), though this difference was not statistically significant (35).

LCBDE is often indicated for patients who require concurrent cholecystectomy and CBD stones management. It offers the advantage of treating both the gallbladder stones and the common bile duct stones in a single-stage procedure. A retrospective study comparing 1-stage management (LCBDE) and 2-stage management (ERCP + laparoscopic cholecystectomy) for patients with gallstones and CBD stones found that the LCBDE group had the shortest operation duration and hospital stay, as well as the lowest long - term postoperative complications, particularly the recurrence rate of CBD stones (36). However, there are also contraindications for LCBDE. Patients with severe inflammation in the biliary area, such as acute cholangitis or pancreatitis, may not be suitable candidates initially, as the inflammation can make the procedure technically difficult and increase the risk of complications. Additionally, patients with complex biliary anatomy, such as those with a history of multiple biliary surgeries or anatomical variations, may require alternative treatment options as LCBDE may be challenging to perform safely in these cases.

Success rates of LCBDE are comparable to EST (24); detailed head-to-head data are presented in Comparative Analysis above. LCBDE achieved a stone-clearance rate of 94.7% (1,810/1,911 patients; pooled proportion 94.7%; 95% CI 93.5%–95.8%) in a recent systematic review and meta-analysis (37). In terms of LCBDE, a high stone clearance rate can be achieved when the procedure is performed by experienced surgeons and appropriate techniques are used.

Another advantage is the preservation of the sphincter of Oddi function. Unlike EST, which may disrupt the sphincter of Oddi, LCBDE can potentially preserve its normal function. Preserving the sphincter of Oddi function may help in maintaining normal bile flow regulation and reducing the long-term risk of complications such as cholangitis and pancreatitis. A study evaluating the long-term risk of pancreatitis and cholangitis after different treatments for gallstone disease found that patients who underwent endoscopic sphincterotomy had a higher risk of these complications compared to those who did not, suggesting the potential benefit of preserving the sphincter of Oddi function (29).

Regarding complication rates, bile leak is a concern in LCBDE. Although the incidence can vary, it is important to ensure proper closure of the common bile duct after stone extraction to minimize this risk. Retained stones are also a potential complication. A study looking at the prevalence of clinically significantly retained common bile duct stones after laparoscopic cholecystectomy found that the prevalence was 1.84% in patients without preoperative evidence of CBD or intrahepatic duct stones, highlighting the importance of thorough stone removal during the procedure (38). However, compared to some other procedures, LCBDE can offer a relatively low complication rate when performed in appropriate patients.

#### Comparative analysis: EST vs. LCBDE

Meta-analyses have been conducted to compare the success rates of EST and LCBDE in terms of stone clearance. A meta - analysis comparing different endoscopic procedures for common bile duct stones, including EST and related techniques, found that for certain stone sizes and conditions, the success rates can be similar. For example, in the treatment of large bile duct stones, both EST-related techniques and LCBDE can achieve relatively high stone clearance rates. However, success could also depend on factors such as the size and location of the stones, as well as the experience of the operator (39). Another study comparing the efficacy of different methods for difficult biliary cannulation, which is often a part of EST, and LCBDE-related procedures showed that in some cases, the success rates may vary. In patients with difficult biliary cannulation, Wang et al. (23) reported that transpancreatic sphincterotomy (TPS) succeeded in achieving selective bile-duct cannulation in 90.3% (65/72) of cases, significantly higher than the 70.8% (46/65) success rate observed with needle-knife sphincterotomy (NKS) (*P* = 0.007). Overall, the success rates of EST and LCBDE are comparable in many clinical scenarios, but careful patient selection and appropriate technique application are crucial.

In terms of short-term complications, both EST and LCBDE have their own profiles. EST is more commonly associated with pancreatitis and bleeding in the short-term. As mentioned earlier, the incidence of post-ERCP pancreatitis in patients undergoing EST can be significant, and factors such as endoscopic biliary stenting can increase this risk (28). Bleeding can also occur during or after EST, especially in patients with coagulation abnormalities or when the procedure is technically challenging.

LCBDE, on the other hand, may be more prone to complications such as bile leak in the short-term. The manipulation of the common bile duct during the procedure can lead to bile leakage if the closure is not proper. In the long-term, EST may lead to papillary dysfunction, which can result in problems such as recurrent biliary obstruction and cholangitis. A long-term follow-up study of patients who underwent EST showed an increased risk of cholangitis and pancreatitis, potentially related to papillary dysfunction (29). LCBDE, if performed successfully with preservation of the sphincter of Oddi function, may have a lower long-term risk of such papillary-related complications.

Cost-effectiveness and hospital stay are important factors to consider when comparing EST and LCBDE. In terms of cost-effectiveness, a study comparing different management strategies for gallstones and CBD stones found that the single-stage LCBDE approach was associated with lower costs compared to two - stage approaches that included preoperative or postoperative ERCP (which often involves EST) (36). This is because LCBDE reduces the need for multiple procedures, hospitalizations, and associated costs. In contrast, EST may sometimes require multiple sessions, especially if stone removal is difficult, which can increase the overall hospital stay and associated costs.

Several factors influence the choice between EST and LCBDE. Stone size is an important factor. For smaller stones, EST may be a more straightforward option as it can often achieve high stone clearance rates with less invasive means. For example, in the treatment of stones ≤ 10 mm, EST-related techniques with appropriate stone extraction tools can be effective (19). However, for larger stones or when there are multiple stones, LCBDE may offer better access for complete stone removal, especially if the anatomy allows for it.

Anatomy also plays a crucial role. Patients with a favorable cystic duct anatomy may be suitable candidates for the transcystic approach in LCBDE. On the other hand, if the anatomy of the ampulla of Vater is distorted or there are other anatomical abnormalities, EST may be more challenging, and LCBDE may be a better alternative. The availability of expertise is another key factor. If a center has experienced endoscopists, EST may be a preferred option. However, if the surgical team has more experience with LCBDE, this may be the chosen approach. Additionally, patient comorbidities and overall health status need to be considered, as some patients may be better suited for the less invasive EST, while others may tolerate the more invasive LCBDE if the potential benefits outweigh the risks.

The two tables below (Tables 1, 2) illustrate the steps and differences between the two surgical procedures, respectively.

**Table 1:** Procedural differences between EST and LCBDE.

EST	LCBDE
1. Duodenoscope inserted orally to locate the ampulla of Vater in the duodenum	1. Laparoscopic ports placed; gallbladder and bile ducts exposed via abdominal exploration
2. Sphincterotome (e.g., needle-knife or pull-type) inserted to incise the sphincter of Oddi	2. Intraoperative cholangiography confirms stone location; approach selected (transcystic or choledochotomy)
3. Stones extracted with balloons or baskets (≤10 mm directly; large stones require adjunctive lithotripsy)	3. Laparoscopic cholangioscopy guides stone extraction with baskets/balloons (large stones fragmented intraoperatively)
4. No suturing required; post-procedure monitoring for bleeding or pancreatitis	4. Choledochotomy closed with sutures and drainage placed; concurrent cholecystectomy performed

EST side highlights “natural orifice access,” “sphincter incision,” and “no abdominal incisions”; LCBDE side emphasizes “laparoscopic port access,” “direct biliary visualization,” and “simultaneous cholecystectomy”; Arrows indicate pathways (EST: Oral → Duodenum → Bile Duct; LCBDE: Abdominal Ports → Abdomen → Bile Duct).

**Table 2:** Comparison (EST vs. LCBDE).

Parameter	EST	LCBDE
Access Route	Transoral via duodenoscope to ampulla of Vater.	Laparoscopic ports (abdominal access) to expose bile ducts.
Key Step	Incision of sphincter of Oddi using sphincterotome (needle-knife or pull-type).	Direct visualization via cholangioscopy; stone extraction via transcystic or choledochotomy approach.
Stone Handling	Balloon/basket extraction; adjuncts (SpyGlass, ESWL) for large/complex stones.	Basket/balloon extraction; intraoperative lithotripsy for large stones.
Concurrent Procedures	Not applicable (separate cholecystectomy if needed).	Simultaneous cholecystectomy possible (single-stage management).
Sphincter Function	Sphincter of Oddi is incised (risk of long-term dysfunction).	Sphincter function preserved (reduces long-term cholangitis/pancreatitis risk).
Major Complications	Post-procedural pancreatitis (9.8% overall), bleeding, rare perforation.	Bile leak, retained stones (1.84% prevalence), wound-related issues.
Best For	Small stones (≤10 mm), high-risk patients, no need for concurrent cholecystectomy.	Large/complex stones (>10 mm), need for concurrent cholecystectomy, favorable cystic duct anatomy.

## Emerging techniques and adjunctive therapies

### Spyglass cholangioscopy: direct visualization and laser lithotripsy

SpyGlass cholangioscopy has emerged as a valuable tool for the management of difficult biliary stones. It allows for direct visualization of the bile ducts, enabling more accurate diagnosis and treatment. In cases where conventional ERCP fails to remove stones, SpyGlass guided laser lithotripsy can be effective. A prospective single center series reported a 94% stone clearance rate using SpyGlass DS peroral cholangioscope-guided laser lithotripsy for complicated biliary stones (27). The technique is relatively safe, with complications such as cholangitis and respiratory distress being reported in a small number of cases, which can usually be managed conservatively. This technique is particularly useful for impacted stones or stones that are difficult to access with traditional methods.

Clinical study has shown that the SpyGlass system was able to provide high-definition endoscopic images, enabling doctors to identify and locate stones more accurately, thereby improving the success rate of treatment (40). Additionally, the SpyGlass system combined with electrohydraulic lithotripsy (EHL) has demonstrated excellent results in treating complex bile duct stones, effectively breaking large stones into smaller pieces, thereby improving the success rate of endoscopic treatment and reducing the need for surgery (41). It should be noted that endoscopic working channel inspections conducted using the SpyGlass system revealed that 5.4% of duodenoscopes were still contaminated with high levels of microorganisms despite being cleaned according to the manufacturer's instructions (42). This suggests limitations of the SpyGlass system in microbial contamination control. The SpyGlass system also has certain limitations in obtaining biopsy samples. Although it can perform precise targeted biopsies under direct visualization, the size limitations of the biopsy forceps may result in insufficient sample quantity and quality, thereby affecting the sensitivity of pathological diagnosis (43). Therefore, combining traditional perspective-guided biopsy may be an effective strategy for improving diagnostic sensitivity. Lastly, the SpyGlass system could cause some post-op complications in certain situations. For example, in patients with bile duct strictures, using the SpyGlass system could lead to complications like acute edematous pancreatitis, although these complications can usually be treated with the right care (44). For these reason, it is necessary to weigh the potential clinical benefits of the SpyGlass system against the possible risks to ensure patient safety and treatment efficacy.

### Extracorporeal shock wave lithotripsy (ESWL): role in large or impacted stones

For stones not amenable to endoscopic removal, extracorporeal shock wave lithotripsy (ESWL) offers a non-invasive fragmentation alternative. It uses shock waves to fragment stones, making them easier to pass or remove. Cholangitisof patients with difficult - to - retrieve CBD STONES, ESWL achieved total CBD clearance in 80.6% of patients (95% CI 71.2%–88.1%) (45). However, factors such as stone size, location, and the presence of cholangitis can affect the success rate. For example, failure of the treatment was more likely in cases of large stones (≥2 cm), incarcerated stones, and pre-endoscopic retrograde cholangiopancreatography cholangitis (45). Complications of ESWL can include pain, bleeding, and pancreatitis, although the overall complication rate is relatively low.

Previously, ESWL achieved complete removal of common bile duct stones in 90% of patients, with a low incidence of complications, with only a few patients developing cholangitis and acute cholecystitis (46). However, obese patients have lower success rates when undergoing ESWL treatment and require more treatment sessions (47). Furthermore, common bile duct diameter, the presence of gallbladder stones, and the maximum size of the stones are considered risk factors for stone recurrence (48). These factors suggest that when selecting ESWL as a treatment option, it is necessary to comprehensively consider the individual differences of patients and the characteristics of the stones. Technically, the efficiency of ESWL is closely related to the type of lithotripter used, as well as the frequency and energy of the shock waves. It was reported that different types of lithotripters were compared, and it was found that the Modulith SLX-F2 had advantages in reducing the incidence of adverse events and the number of treatments (49). Additional study has shown that using saline infusion improved the fragmentation of stones, resulting in higher treatment success rates (50).

The efficacy and safety of ESWL still require further study compared with other treatment methods, although a favorable outcome has been demonstrated in the treatment of CBD stones. For instance, ESWL has a lower complete stone clearance rate compared to laser lithotripsy, but a relatively lower complication rate (51). Accordingly, individualized treatment plans should be developed based on the specific circumstances of each patient and the characteristics of the stones. Continued exploration of the efficacy of ESWL in different patient populations is warranted, along with comparisons with other treatment modalities, to provide more comprehensive treatment strategy guidance.

### Medical dissolution therapy: ursodeoxycholic acid (UDCA) for specific stone types

Medical dissolution therapy with ursodeoxycholic acid (UDCA) is an option for specific types of CBD STONES, particularly cholesterol stones. UDCA works by reducing the cholesterol saturation of bile, potentially leading to the dissolution of stones. A randomized trial comparing the CBD recurrence rate after bile duct stone removal between patients given UDCA and the untreated group suggested that UDCA may be a novel treatment strategy to prevent the recurrence of CBD stones, with a recurrence rate of 6.6% in the UDCA group compared to 18.6% in the untreated group, although the difference was not statistically significant (absolute difference −12.0%; 95% CI −25.9% to 1.9%; *P* = 0.171) (52). However, the effectiveness of UDCA may depend on factors such as stone size and composition, and it may take a long time to achieve significant results.

**Figure 1:**
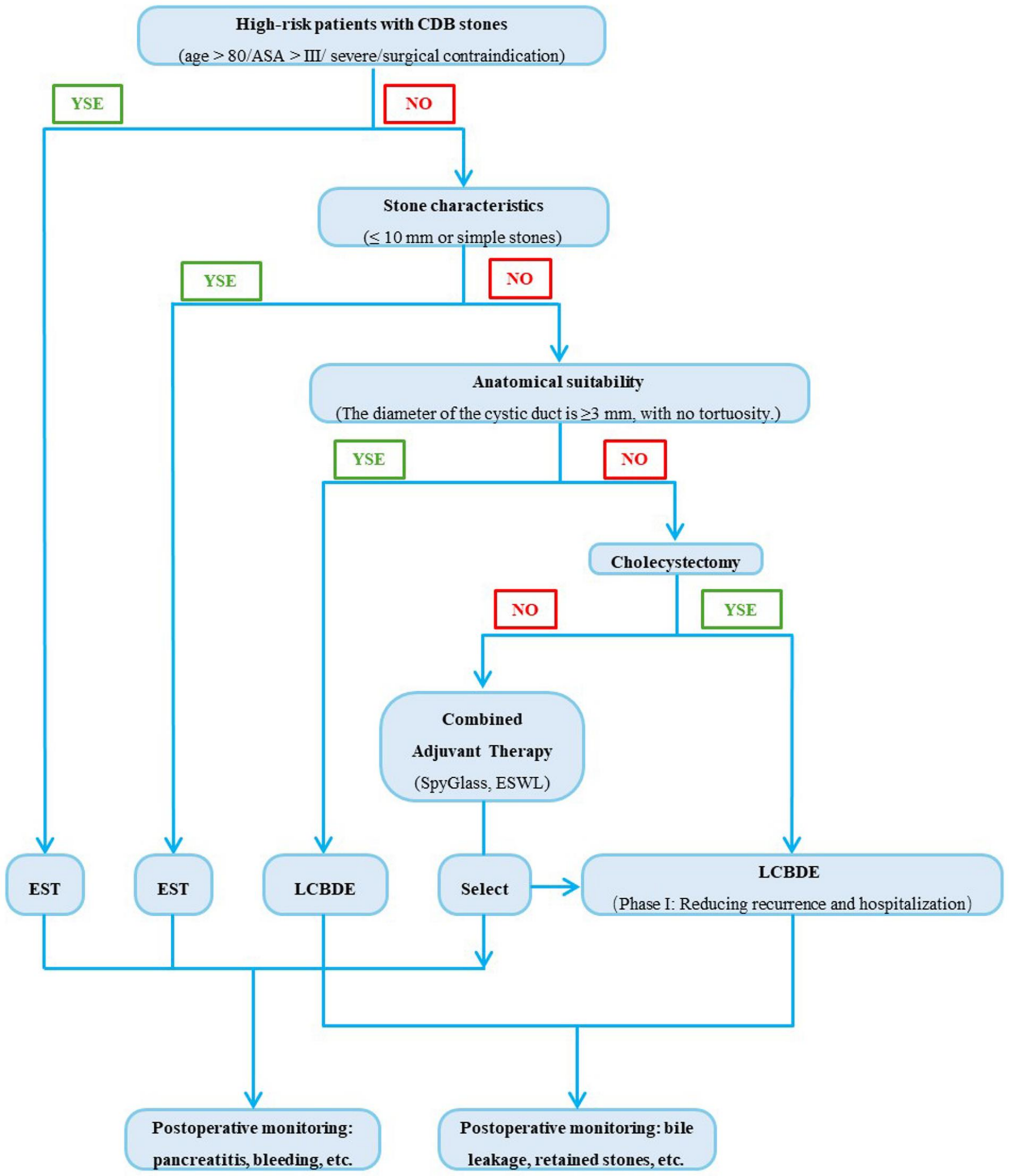
CBD tones management: decision tree for EST vs. LCBDE selection. Step by step guidance on surgical procedure selection based on key factors such as patient risk stratification, stone characteristics, and anatomical conditions. 1) Assess Patient Risk Status. If patient is high-risk (e.g., advanced age, severe comorbidities, poor surgical tolerance), prioritize EST (supported by manuscript evidence that EST is preferable for high-risk surgical candidates). If patients are low/medium-risk, proceed to evaluate stone characteristics. 2) Evaluate Stone Characteristics. For small (≤10 mm) and simple stones, prioritize EST (EST achieves >90% clearance for stones ≤ 10 mm). For large (>10 mm) or complex stones (e.g., multiple, impacted, or associated with strictures), proceed to assess anatomical suitability. 3) Assess Anatomical Suitability. If cystic duct anatomy is favorable (≥3 mm diameter, non-tortuous), prioritize LCBDE via transcystic approach. If cystic duct anatomy is unfavorable: If concurrent cholecystectomy is needed, prioritize LCBDE (single-stage management reduces recurrence and hospital stay). If no cholecystectomy is needed, base the decision on institutional expertise (EST with adjuncts like SpyGlass or mechanical lithotripsy vs. LCBDE). 4) Post-Procedural Monitoring. For EST: Monitor for post-procedural pancreatitis (9.7% incidence) and bleeding. For LCBDE: Monitor for bile leak and retained stones.

To facilitate bedside decision-making, we propose a concise algorithm (Figure 1) that integrates patient risk profile, stone size, and anatomical complexity.

## Challenges and future directions

### Unresolved issues: long-term outcomes of EST related sphincterotomy

The long-term outcomes of EST - related sphincterotomy remain an area of concern. While EST is an effective short - term treatment for CBD STONES, there are potential long-term complications. One of the main issues is the risk of post-EST pancreatitis, which can occur in the long - term and may lead to chronic pancreatitis in some cases. In a study of pediatric patients who underwent EST, long - term complications (>30 days) developed in 6.1% (95% CI 2.9%–11.0%) of patients, including cholangitis with or without bile duct stone and minor papilla restenosis (53). Another concern is the impact on the sphincter of Oddi function, which may lead to reflux of duodenal contents into the biliary tree, increasing the risk of recurrent cholangitis. More research is needed to fully understand these long - term outcomes and develop strategies to mitigate the risks.

### Standardization of LCBDE training and accessibility

The standardization of LCBDE training is crucial for ensuring the quality and safety of the procedure. Currently, there is a lack of uniform training guidelines, which may lead to variations in the skills and outcomes of surgeons performing LCBDE. A study on the training of bronchoscopy skills in pulmonology residents found that a standardized one-day simulation-based training course led to rapid improvement of basic bronchoscopy skills (54). Similar standardized training programs could be developed for LCBDE. Additionally, improving the accessibility of LCBDE is important, especially in regions with limited resources. This may involve training more surgeons in the technique and ensuring the availability of the necessary equipment.

### Innovations: hybrid approaches

**Figure 2:**
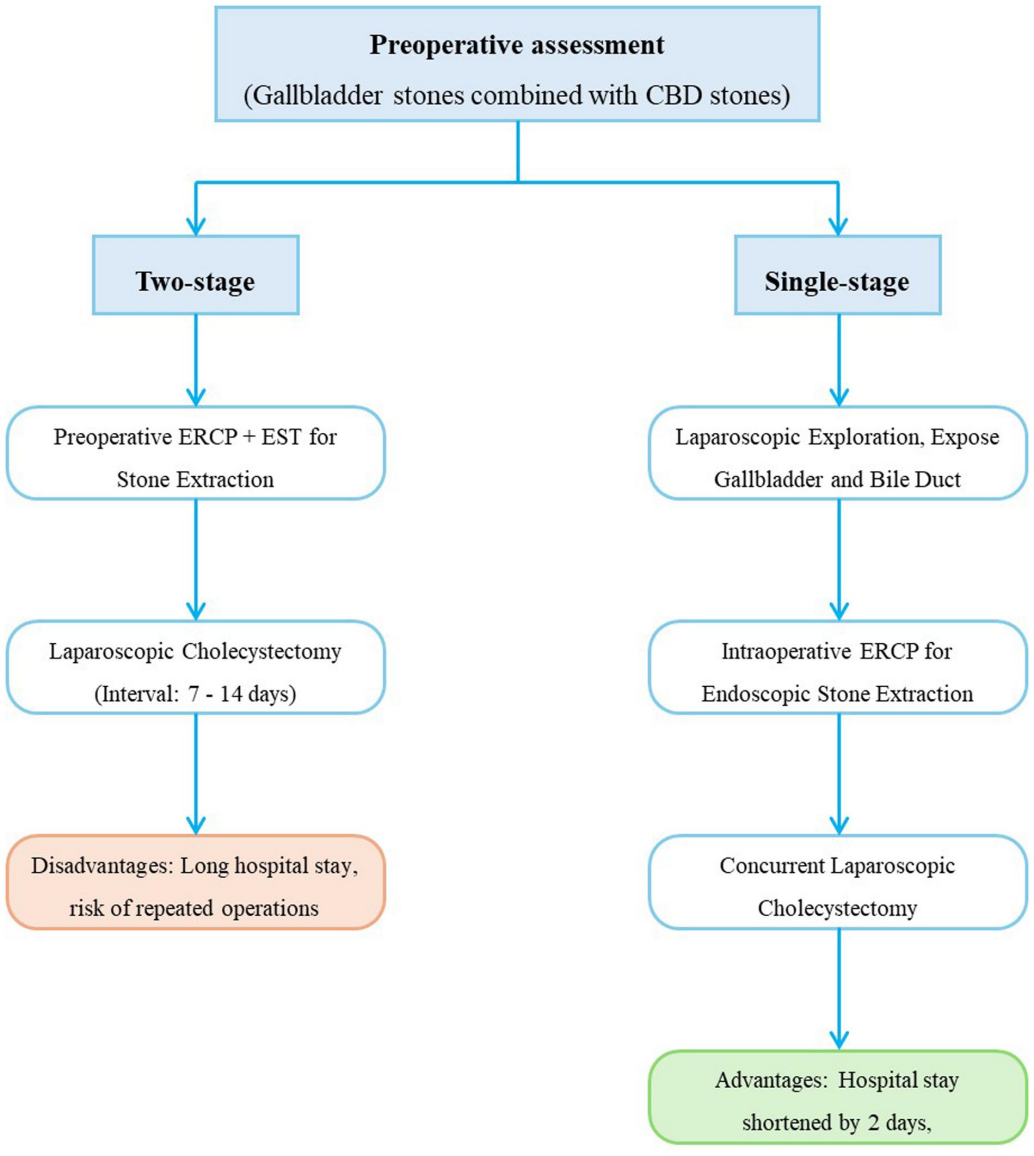
Intraoperative ERCP + laparoscopic cholecystectomy. 1) Preoperative confirmation of CBD stones via imaging (MRCP/EUS). 2) Laparoscopic exploration to expose gallbladder and bile duct anatomy. 3) Intraoperative ERCP performed to clear CBD stones via endoscopic techniques (EST + balloon/basket extraction). 4) Concurrent laparoscopic cholecystectomy to remove gallbladder stones. 5) Postoperative outcomes: Shorter hospital stays (−2.04 days vs. two-stage protocols) and lower pancreatitis risk (RR 0.37).

Hybrid approaches, such as intraoperative ERCP, are emerging as innovative strategies for the treatment of CBD stones. Intraoperative ERCP combines the advantages of endoscopic and surgical techniques, allowing for real-time diagnosis and treatment of CBD stones during laparoscopic cholecystectomy. A meta-analysis comparing single-stage intraoperative ERCP combined with laparoscopic cholecystectomy vs. preoperative ERCP followed by laparoscopic cholecystectomy found that the intraoperative ERCP group had a shorter length of hospital stay (mean difference in length of stay −2.04 days; 95% CI −2.75 to −1.33; *P* < 0.001), lower overall morbidity (RR 0.55; 95% CI 0.35–0.85; *P* = 0.007), and a lower rate of postoperative pancreatitis (RR 0.37; 95% CI 0.18–0.78; *P* = 0.009) (7). However, these procedures require a high level of expertise and coordination between the surgical and endoscopic teams. Figure 2 summarizes the comparison between single-stage intraoperative ERCP combined with laparoscopic cholecystectomy and preoperative ERCP followed by laparoscopic cholecystectomy.

### Artificial intelligence in stone characterization and procedural planning

**Figure 3:**
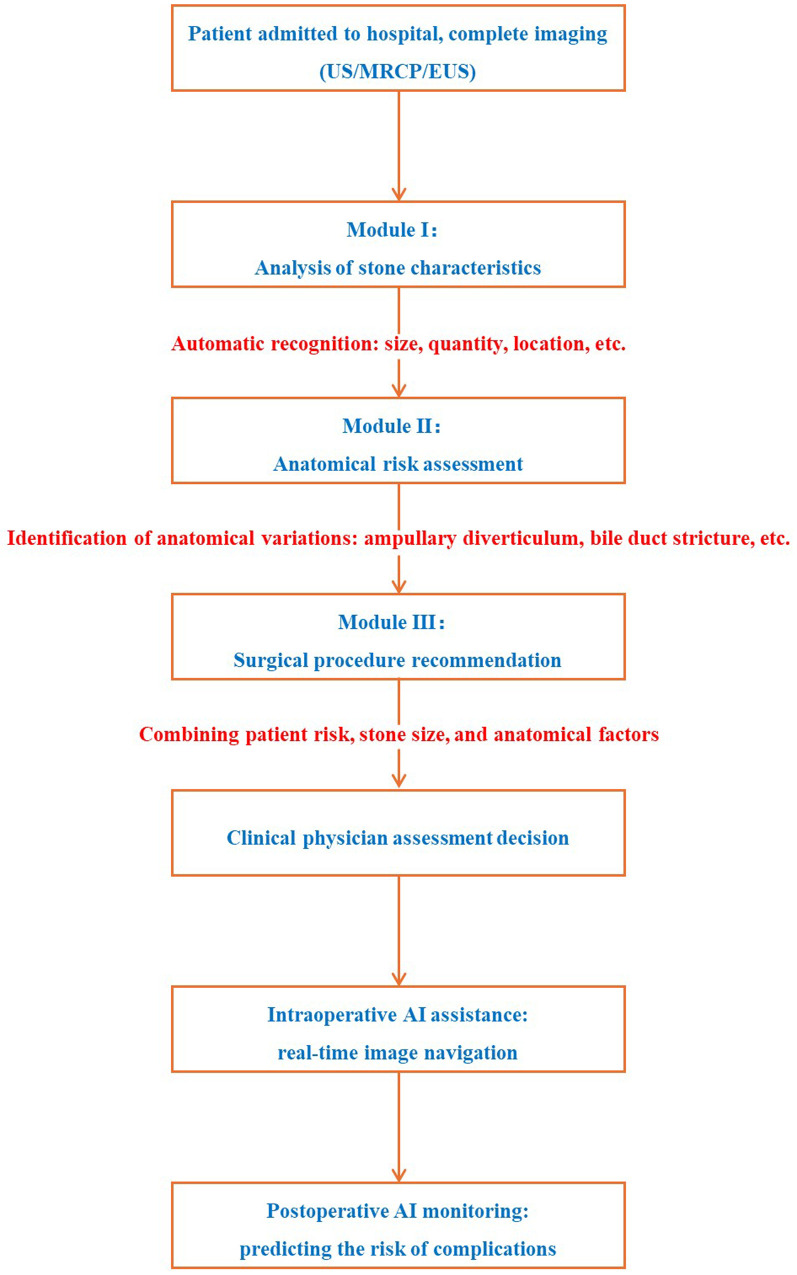
AI-Assisted workflow for CBD stones management. 1) Preoperative Phase: AI analyzes imaging (MRCP/EUS) to characterize stones (size, location, composition) and detect anatomical variants (e.g., periampullary diverticulum). AI integrates patient data (age, comorbidities) to recommend optimal procedure (EST/LCBDE/hybrid). 2) Intraoperative Phase: AI-assisted real-time navigation (e.g., cholangioscopy image fusion) to enhance stone detection and extraction accuracy. 3) AI predicts complication risk (e.g., pancreatitis for EST, bile leak for LCBDE) to guide monitoring protocols. AI advantages: improve the accuracy of stone detection, optimize.

Artificial intelligence (AI) has the potential to revolutionize the management of CBD stones. In stone characterization, AI can analyze imaging data to accurately identify the type, size, and location of stones, providing more detailed information for treatment planning. For example, in other medical fields, AI based algorithms have been used to analyze radiographs to predict the risk of hip dislocation following total hip arthroplasty (55). In procedural planning, AI can assist in determining the most appropriate treatment approach based on patient characteristics and stone features. It can also potentially be used to improve the training of surgeons by providing virtual reality simulations of procedures. However, the implementation of AI in CBD stones management requires further research to validate its accuracy and effectiveness. Figure 3 illustrates the process of managing chronic cholangitis stones based on artificial intelligence.

## Discussion

EST and LCBDE are complementary. EST suits small stones or high-risk patients; LCBDE excels for large or complex stones. Selection should integrate stone characteristics, patient comorbidities, and institutional expertise. It offers the advantage of direct visualization of the biliary tree and can potentially achieve a higher rate of complete stone clearance in some cases. The choice between the two techniques should be based on a comprehensive assessment of the patient's condition, including stone characteristics, comorbidities, and the expertise of the medical team.

Personalized treatment is of utmost importance in the management of CBD STONES. Patient characteristics, such as age, comorbidities, and the presence of anatomical variations, can significantly influence the choice of treatment. For example, in patients with severe comorbidities, a less invasive approach like EST may be preferred, while in younger and healthier patients with larger stones, LCBDE may offer better long-term outcomes. Institutional expertise also plays a crucial role. Centers with experienced endoscopic teams may be more inclined to perform EST, while those with skilled laparoscopic surgeons may opt for LCBDE. By tailoring the treatment to the individual patient and the capabilities of the institution, better outcomes can be achieved.

There is a clear need for randomized trials comparing the long-term outcomes and cost-effectiveness of different treatment strategies for CBD STONES. Current evidence is often based on small - scale studies or retrospective analyses, which may have limitations in terms of bias and generalizability. Randomized trials would provide more robust data on the long-term efficacy, safety, and cost-effectiveness of EST, LCBDE, and emerging techniques. For example, a randomized trial comparing the long-term outcomes of EST and LCBDE could help determine which technique is more suitable for different patient populations in terms of recurrence rates, quality of life, and healthcare costs. Such trials would also help in establishing evidence-based guidelines for the management of CBD STONES.

## Key research gaps and recommendations

### Head-to-head RCTs in low-resource settings

High-quality randomized trials comparing EST and LCBDE are still lacking in low- and middle-income countries where equipment, expertise, and patient profiles differ markedly. Multi-center RCTs comparing EST and LCBDE in low-resource settings are urgently needed to close the global evidence gap.

### Cost-effectiveness of hybrid procedures

Formal economic evaluations (cost-utility or cost-minimization analyses) of single-stage intraoperative ERCP vs. sequential treatment strategies are limited; no study has incorporated long-term quality-adjusted life years (QALYs). Formal cost-utility analyses should be embedded in upcoming hybrid-procedure trials to clarify the economic value of single-stage intraoperative ERCP.

### AI implementation barriers

Although AI shows promise for stone characterization and procedural planning, real-world validation, data privacy, regulatory approval, and surgeon training hurdles remain largely unaddressed. Prospective implementation studies of AI-driven stone characterization tools must follow, focusing on real-world validation, clinician training, and data-security frameworks.

### Long-term sphincter function after EST vs. LCBDE

Prospective cohorts with ≥10-year follow-up are needed to quantify rates of papillary stenosis, recurrent cholangitis, and pancreatitis in diverse populations. Long-term, 10-year prospective registries should be established to definitively quantify sphincter-preservation benefits and late biliary sequelae after EST vs. LCBDE.

### Optimal management of anatomically complex cases

Comparative trials in patients with prior biliary surgery, altered anatomy, or concomitant intra-hepatic calculi are absent; current evidence relies on small retrospective series. Dedicated comparative trials for patients with complex or postsurgical anatomy are essential to refine patient-specific algorithms and optimize outcomes in this high-risk cohort.
